# eHealth Literacy in People Living with HIV: Systematic Review

**DOI:** 10.2196/publichealth.9687

**Published:** 2018-09-10

**Authors:** Hae-Ra Han, Hyejeong Hong, Laura E Starbird, Song Ge, Athena D Ford, Susan Renda, Michael Sanchez, Jennifer Stewart

**Affiliations:** 1 School of Nursing The Johns Hopkins University Baltimore, MD United States; 2 Center for Cardiovascular and Chronic Care The Johns Hopkins University Baltimore, MD United States; 3 Department of Natural Sciences University of Houston-Downtown Houston, TX United States; 4 Department of Nursing University of Houston-Downtown Houston, TX United States

**Keywords:** eHealth literacy, HIV, systematic review, mobile phones

## Abstract

**Background:**

In the era of eHealth, eHealth literacy is emerging as a key concept to promote self-management of chronic conditions such as HIV. However, there is a paucity of research focused on eHealth literacy for people living with HIV (PLWH) as a means of improving their adherence to HIV care and health outcome.

**Objective:**

The objective of this study was to critically appraise the types, scope, and nature of studies addressing eHealth literacy as a study variable in PLWH.

**Methods:**

This systematic review used comprehensive database searches, such as PubMed, EMBASE, CINAHL, Web of Science, and Cochrane, to identify quantitative studies targeting PLWH published in English before May 2017 with eHealth literacy as a study variable.

**Results:**

We identified 56 unique records, and 7 papers met the eligibility criteria. The types of study designs varied (descriptive, n=3; quasi-experimental, n=3; and experimental, n=1) and often involved community-based settings (n=5), with sample sizes ranging from 18 to 895. In regards to instruments used, 3 studies measured eHealth literacy with validated instruments such as the eHealth Literacy Scale (eHEALS); 2 studies used full or short versions of Test of Functional Health Literacy in Adults, whereas the remaining 2 studies used study-developed questions. The majority of studies included in the review reported high eHealth literacy among the samples. The associations between eHealth literacy and health outcomes in PLWH were not consistent. In the areas of HIV transmission risk, retention in care, treatment adherence, and virological suppression, the role of eHealth literacy is still not fully understood. Furthermore, the implications for future research are discussed.

**Conclusions:**

Understanding the role of eHealth literacy is an essential step to encourage PLWH to be actively engaged in their health care. Avenues to pursue in the role of eHealth literacy and PLWH should consider the development and use of standardized eHealth literacy definitions and measures.

## Introduction

HIV is a major global health issue with an estimated 36.7 million people living with HIV (PLWH) worldwide [1]. In the United States, 1.1 million individuals are estimated to have HIV [2]. With the advent of antiretroviral therapy (ART), HIV has become a chronic condition requiring self-management, including the adherence to ART and keeping regular HIV care appointments [3]. However, PLWH often do not adhere to their treatment regimen; only 30% are ART adherent to the point of achieving viral suppression [4].

eHealth, “a medical and public health practice supported by a Web-based platform,” is a popular innovation in self-management of chronic conditions and includes mobile phones, tablet computers, and personal computers [5]. Web-based electronic communication technology is a relatively new source of health information that requires a new set of health literacy skills. Internet access is now nearly unlimited with 89% of US adults using the internet to access health information and gain social support [6]. This eHealth not only increases the access to health information but also expands social support and coping strategies by linking people together largely through a network of commercial, educational, and governmental websites as well as social media [7]. The utility of eHealth as an effective health communication and educational tool for self-management of chronic conditions has already been demonstrated [8]. In addition, evidence indicates that eHealth interventions offer great promise to promote care across the HIV treatment cascade, including prevention [9], medication adherence [10,11], and quality of life [12].

eHealth literacy refers to one’s ability “to seek, find, understand, and appraise health information from electronic sources and apply the knowledge gained to address or solve a health problem” [13]. In this era of eHealth, PLWH represent an important population in which to intervene on eHealth literacy as electronic health sources is a more feasible and cost-effective means to improve the adherence to HIV care continuum, treatment outcomes, and promote health for PLWH [14]. Hence, this study aims to critically appraise the types, scope, and nature of studies designed to address eHealth literacy as a study variable in PLWH.

To the best of our knowledge, this is the first systematic review to address eHealth literacy in PLWH. Although previous systematic reviews have addressed eHealth literacy in college students [15], underserved populations [16], or older adults [17], eHealth literacy tools [18], Web-based health literacy interventions [19], computer-based interventions and applications [20], and eHealth policy issues [21], none were focused on PLWH. We aim to explain the definitions of eHealth literacy used in each study, describe theoretical and measurement approaches pertaining to eHealth literacy, and evaluate the study findings on eHealth literacy in association with target behavior or health outcomes in PLWH to identify gaps and areas for potential future research.

## Methods

### Review Design

We conducted a systematic review of quantitative evidence designed to assess eHealth literacy as a study variable in PLWH. Owing to the heterogeneity relative to study designs and statistical analysis approaches among the included studies, we synthesized the study findings rather than conducting a meta-analysis.

### Study Eligibility

Studies were screened to assess their relevance for our review. Specifically, the following inclusion criteria were used papers that used a quantitative study design (including descriptive, correlational, quasi-experimental, or experimental); papers including eHealth literacy as a study variable; and papers including participants with HIV or AIDS. Our initial search was not limited by the age of study participants or sex to maximize the breadth of the study findings. In addition, we included any study that reported quantitative findings relevant to the review question. Studies from around the globe were included, as were studies conducted in various settings, including community or health system settings.

Notably, only studies written in English were included. Studies were excluded if full-texts were unavailable (ie, conference abstracts), they were not quantitative designs, or they reported protocol only with no measured outcomes.

### Search and Identification Process

In consultation with a medical librarian, peer-reviewed journal papers were searched systematically in PubMed, EMBASE, CINAHL, Scopus, Web of Science, and Cochrane databases using variations of MeSH terms—methodological interest (ie, measurement of eHealth literacy as a study variable), population of interest (ie, PLWH), and study design of interest (ie, quantitative) to identify relevant papers published in English before April 27, 2017. In addition, a manual search of reference lists in selected papers was completed. Multimedia Appendix 1 provides a full search strategy for the database searches. Papers and abstracts were excluded if they did not address the population, design, or variable of interest.

### Data Extraction and Quality Assessment

At the conclusion of the study selection process, 1 reviewing author extracted data from the studies using a standard template. The initial data extraction captured both the study characteristics (eg, setting, participants, type of study design, and eHealth literacy measure) and key findings from each study. In addition, other team members reviewed the studies and extracted data relating to key findings. Extracted findings were compared and discussed until all discrepancies were resolved.

We assessed the rigor of the underlying evidence base for the review by developing an overview of key methodological characteristics, including the study design, sample size and strategy, study setting, and year of publication. No studies were excluded on the basis of the quality assessment. Rather, the quality assessment process was conducted independently by 2 raters using the Joanna Briggs Institute quality appraisal tools based specifically on study designs, randomized controlled trials (RCTs) [22], quasi-experimental [22], and cross-sectional [23] studies to identify strengths and weaknesses in study methodologies and guide the interpretation and assessment of study findings.

## Results

### Selection of Studies

**Figure 1:**
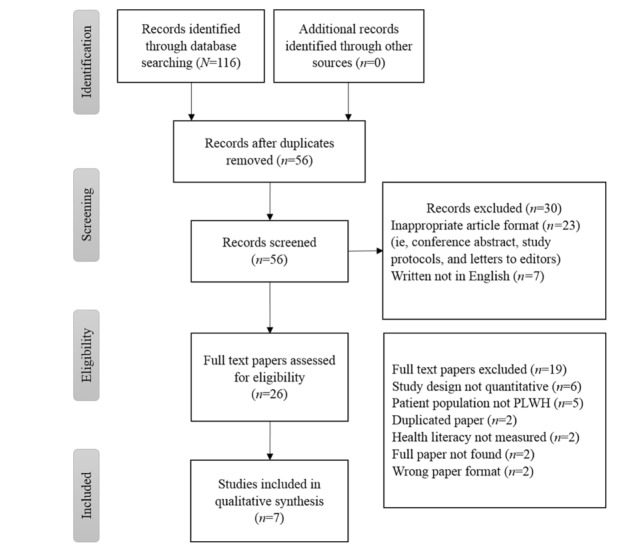
Literature review flowchart.

Figure 1 presents a detailed outline of the paper selection process. Our initial database search in April 2017 resulted in 116 citations. After removing duplicates, 56 titles with abstracts were reviewed independently for relevance by 2 authors (among HH, LES, and SG). The third author resolved conflicts in the inclusion of papers. Overall, 26 papers passed on to the next full-text review process. Of 26 full-text papers that were reviewed independently by 2 authors, 7 were deemed eligible. Reasons for exclusion included study design not quantitative (n=6), patient population not PLWH (n=5), duplicated paper (n=2), eHealth literacy not measured (n=2), full paper not found (n=2), and wrong format of paper (ie, not a journal paper; n=2).

### Quality Assessment: Characterizing the Evidence Base

Overall, the studies appraised in this review achieved, at least, the assessment criteria, but the quality varied across individual studies. Although 1 RCT scored 8 of 13 [24] and 1 of 3 quasi-experimental studies scored 6 of 9 [25], they exhibited strengthened validity of causal inferences by comparing the control and intervention groups. In addition, 2 quasi-experimental studies scored 6 of 9 [26,27] and lacked a comparison group to determine pre-post intervention effects. One cross-sectional study earned a perfect score of 8 of 8 [28]; the remaining 2 earned 4 and 6, respectively [29,30]; potential confounding factors were not identified in these 2 studies. In addition, 2 of 7 studies did not use a validated standard measure of eHealth literacy but collected participants’ basic literacy skills [24,29].

Furthermore, an interrater agreement rate was calculated [31]. The resulting statistic indicated substantial agreement (average interrater agreement rate, 69%) [32]. For items where discrepancies occurred between raters, we resolved them by interrater discussion. Table 1 shows consensual scores of the quality assessment.

**Table 1:** Quality assessment.

Study items		Siedner et al [24] (n=8)	Ownby et al [26] (n=6)	Robinson et al [27] (n=6)	Woods et al [25] (n=6)	Blackstock et al [28] (n=8)	Kim et al [29] (n=4)	Krishnan et al [30] (n=6)
**Randomized controlled trial**								
	1. Was true randomization used for assignment of participants to treatment groups?	✔						
	2. Was allocation to treatment groups concealed?							
	3. Were treatment groups similar at the baseline?							
	4. Were participants blind to treatment assignment?							
	5. Were those delivering treatment blind to treatment assignment?							
	6. Were outcomes assessors blind to treatment assignment?							
	7. Were treatment groups treated identically other than the intervention of interest?	✔						
	8. Was follow-up complete and, if not, were differences between groups in terms of their follow-up adequately described and analyzed?	✔						
	9. Were participants analyzed in the groups to which they were randomized?	✔						
	10. Were outcomes measured in the same way for treatment groups?	✔						
	11. Were outcomes measured in a reliable way?	✔						
	12. Was appropriate statistical analysis used?	✔						
	13. Was the trial design appropriate, and any deviations from the standard randomized controlled trial design (individual randomization, parallel groups) accounted for in the conduct and analysis of the trial?	✔						
**Quasi-experimental studies**								
	1. Is it clear in the study what is the “cause” and what is the “effect” (ie, there is no confusion about which variable comes first)?		✔	✔	✔			
	2. Were the participants included in any comparisons similar?				✔			
	3. Were the participants included in any comparisons receiving similar treatment or care, other than the exposure or intervention of interest?							
	4. Was there a control group?				✔			
	5. Were there multiple measurements of the outcome both pre and post the intervention or exposure?		✔	✔				
	6. Was follow-up complete and, if not, were differences between groups in terms of their follow-up adequately described and analyzed?		✔	✔				
	7. Were the outcomes of participants included in any comparisons measured in the same way?		✔	✔	✔			
	8. Were outcomes measured in a reliable way?		✔	✔	✔			
	9. Was appropriate statistical analysis used?		✔	✔	✔			
**Cross-sectional studies**								
	1. Were the criteria for inclusion in the sample clearly defined?					✔	✔	✔
	2. Were the study subjects and the setting described in detail?					✔	✔	✔
	3. Was the exposure measured in a valid and reliable way?					✔		✔
	4. Were objective, standard criteria used for measurement of the condition?					✔		✔
	5. Were confounding factors identified?					✔	✔	
	6. Were strategies to deal with confounding factors stated?					✔		
	7. Were the outcomes measured in a valid and reliable way?					✔		✔
	8. Was appropriate statistical analysis used?					✔	✔	✔

### Overview of Studies Included in the Review

Tables 2 and 3 summarize the main characteristics of studies included in this review. All 7 included studies were published from 2010 to 2016. Of these, 4 studies were conducted in the United States [25-28], 2 in Uganda [24,29], and 1 in Peru [30]. Various study designs used were cross-sectional [28-30], quasi-experimental [25-27], and RCT [24]. Of note, 2 studies identified a theoretical or conceptual framework used in their research [24,26].

Study participants were recruited from a variety of settings as follows: community-based HIV/AIDS organizations [26,28-30], HIV hospital settings [24,27], and both community-based and hospital settings [25]. Overall, HIV-infected adults aged >18 years were included; 1 study included only people who had advanced immunosuppression (CD4^+^ [cluster of differentiation 4] T-cell count <350 cells/mm^3^ and taking ARTs for, at least, 4 years) [29], 1 included women only [28], and 1 involved men who have sex with men and transgender women [30]. The sample sizes ranged from 18 to 895.

Among studies that included women, most had a majority of female participants (56%-100%) but 2 [25,26] included only 9% and 29% of females in their study samples, respectively. Studies in the United States tended to include a large proportion of African American or black (57%-63%) participants [25-28] in which more than half (54.3%) of the study sample was Caucasian. All but 1 study [30] reported low educational levels with 37%-65% of participants having less than high school education. The baseline access to mobile phones, computers, and the internet was fairly high among participants in the United States, Uganda, and Peru. In the United States, 87.3%-88.9% used a smartphone, [25,28], 58.7%-88.9% used a home computer or tablet [25,28], 72.2% had regular access to the internet [27], and 66.7% used the internet daily [25]. Similarly, in Uganda, 81.8%-82.8% of study participants owned a mobile phone [24,29]. Krishnan et al reported that 59.6% of participants in Peru had access to a standard cell phone, 30.1% had access to a smartphone, 37.3% used landlines, and 35.4% accessed a laptop or desktop computer [30].

### Definition and Assessment of eHealth Literacy

In this review, 5 of 7 studies defined eHealth literacy. Most studies [25-28] defined eHealth literacy as the capacity to find, process, understand, and apply health information to make appropriate health decisions. Blackstock et al [28] specified that this information must come from an electronic source. Kim et al [29] simply defined health literacy as the ability to read and write.

In addition, 3 studies conducted in the United States [25,27,28] measured eHealth literacy using the eHealth Literacy Scale (eHEALS), a self-evaluation tool comprising 8 items with a 5-point Likert scale. eHEALS measures the participants’ level of knowledge, comfort, and skills in utilizing the internet or electronic health information to solve health problems [33]. In addition to assessing the ability to utilize internet-based health information using eHEALS, Woods et al [25] determined participants’ general literacy, numeracy levels, and HIV-associated knowledge using a battery, including the Test of Online Pharmacy Skills (TOPS), Test of Online Health Records Navigation (TOHRN), Rapid Estimate of Adult Literacy in Medicine), HIV Knowledge 18, Expanded Numeracy Scale, Short Assessment of Health Literacy, Test of Functional Health Literacy in Adults (TOFHLA) reading comprehension, and Newest Vital Sign.

Moreover, Ownby et al [26] used the full-length version of TOFHLA [34] to measure basic reading and numeracy abilities to understand the verbal and written information commonly used in actual health care settings. Krishnan et al [30] used a short version of TOFHLA [35] in Spanish for screening patient literacy levels in health care settings in Peru. In Uganda, Siedner et al [24] and Kim et al [29] evaluated the feasibility and effect of a mobile phone-based short message service (SMS) text message intervention on the adherence to HIV treatment. eHealth literacy was assessed by study-tailored questions by asking participants to read a full sentence in their local language at enrollment; for example, “Are you able to read and/or write?” along with mobile phone availability [24,29].

**Table 2:** Overview of included studies.

Study	Study design, sample size, and setting	Study purpose	Study framework	Sample characteristics	Definition of eHealth literacy
Blackstock et al, 2016 [28]	Cross-sectional, N=63, February-April, 2014; 6 community-based organizations providing social and clinical services to people living with HIV	To examine the relationship between eHealth literacy and HIV transmission risk behaviors in internet-using women with HIV	No study framework reported	100% female; median age, 49 (IQR^a^ 44-54) years; 54.0% (34/63) non-Hispanic black; 36.5% (23/63) Hispanic; 38.1% (24/63) <high school education; 85.7% (54/63) prescribed ART^b^; 87.3% (55/63) owned a cell phone; 58.7% (37/63) had a computer or tablet	“The ability to find, under-stand, & evaluate health information from electronic sources and apply this information to a specific health problem” (Norman and Skinner, 2006 [13])
Kim et al, 2015 [29]	Cross-sectional, June 2012-August 2013, N=895, AIDS Support Organization	To determine the proportion of people living with HIV who are literate and also use mobile phones in rural Uganda	No study framework reported	76.4% (684/895) female; median age, 44 (IQR 44-50) years; 65% (581/895) <high school education; median time on HIV medications, 6.8 (IQR 5.8-7.7) years; 82.8% (741/895) owned a mobile phone; 73.0% (653/895) can read and write	Ability to read and write
Krishnan et al, 2015 [30]	Cross-sectional, N=359, no specified date, 3 sites at 2 nongovernmental organizations providing health care	To examine the use of communication technology and acceptance of mHealth among HIV-infected Peruvian men who have sex with men and TGW^c^ to gauge the feasibility of an mHealth-enabled HIV-risk reduction program	No study framework reported	77.7% (279/359) male; 13.3% (48/359) TGW; mean age, 34 (SD 8.11) years; 2.2% (8/359) <high school education; 53.3% (131/246) completed college; 87.2% (313/359) currently on ART; 59.6% (214/359) had access to a standard cell phone; 30.1% (108/359) had access to a smartphone; 37.3% (134/359) used landlines; 35.4% (127/359) accessed a laptop or computer	Definition of eHealth literacy not reported
Ownby et al, 2012 [26]	Quasi-experimental, N=124, May 2010-December 2011, Urban and suburban HIV clinics	To evaluate whether an Information-Motivation-Behavioral Skills Model–based electronic intervention can improve health literacy and medication adherence	Information-Motivation-Behavioral Skills model	29% female (36/124); mean age, 47.1 (SD 8.69) years; 63% (78/124) black; 37% (46/124) <high school education; mean, 11.6 (SD 7.18) years on ART; mean Test of Functional Health Literacy in Adults score, 88.48 (SD 14.16)	“The degree to which individuals have the capacity to obtain, process, & understand basic health information & services needed to make appropriate health decisions” (Nielsen-Bohlman et al, 2004 [36])
Robinson et al, 2010 [27]	Quasi-experimental, N=18, July, 2008, HIV-positive care center in a hospital setting	To determine if computer skills and internet health educational intervention will improve the perceived knowledge of internet health resources and confidence using the internet for health questions	No study framework reported	55.6% (10/18) female; mean age, 46 (range 34-69) years; 61.1% (11/18) African American; 27.8% (5/18) Caucasian; 44.4% (8/18) high school education or less; 72% (13/18) have regular internet access; 23% (3/13) sought health information in the internet in the past 3 months	The “capacity to acquire, understand & use information in ways which promote & maintain good health”
Siedner et al, 2015 [24]	Experimental, N=385, HIV clinic of the Mbarara Regional Referral Hospital	To identify predictors of uptake of a mHealth app and evaluate the efficacy of various short message service text message formats to optimize the confidentiality and accessibility	Concepts derived from the Technology Acceptance Model and the Unified Theory of Technology Acceptance and Use of Technology	65.2% (251/385) female; median age 32 (IQR 26-39) years; 62.4% (240/385) primary education or less; 67.5% (260/385) could read a complete sentence; 81.8% (315/385) had a mobile phone	Definition of eHealth literacy not reported
Woods et al, 2016 [25]	Cross-sectional, N=67, neuroAIDS research center, which recruits from local HIV clinics and community-based organizations	To evaluate the effects of HIV-associated neurocognitive disorders on 2 internet-based tests of health care management	No study framework reported	9.0% (6/67) female; 68.7% (46/67) HIV+ and 31.3% (21/67) HIV- mean age 45.5 (SD 9.2) years; 53.7% (36/67) Caucasian; 19.4% (13/67) Hispanic; mean education level 13.2 (SD 2.5) years; 95.7% (44/46) prescribed ART; 86.6% (58/67) use a home computer; 76.1% (51/67) own a smartphone; 67.2% (45/67) use the internet daily	“The capacity to obtain, communicate, process, & understand basic health information & services to make appropriate health decisions” (Patient Protection & Affordable Care Act, 2010 [37])

^a^IQR: interquartile range.

^b^ART: antiretroviral therapy.

^c^TGW: transgender women.

### Characteristics of eHealth Literacy Among People Living With HIV

Overall, varying scales with differing scoring systems were used to determine the level of eHealth literacy. High eHealth literacy scores among PLWH ranged from 52.4% to 87% in study samples with the majority of studies finding high eHealth literacy among 65%-80% of participants. Such a wide variance arose because high literacy was defined differently in each study, ranging from the ability to read a complete sentence [24] to a TOFHLA score >75 [26], and an eHEALS score greater than the median [28]. Kim et al [29] simply asked about the ability to read and write and reported on differences in participant demographic characteristics by literacy; they found that men are more likely to be literate and use a cell phone than women, AOR 2.81 (95% CI 1.83-4.30), and employed participants are more likely to be literate and use a cell phone than those with no income, AOR 2.35 (95% CI 1.23-4.49).

The acceptability of eHealth interventions was measured in 3 studies [27,29,30]. Nearly all (91.7%) patients with high eHealth literacy supported their providers’ use of SMS text messaging communication for reminders or to check health status in contrast to only 38.8% of PLWH who were not literate or did not own a cell phone (*P*<.001) [29]. Daily electronic medication adherence reminders were preferred over weekly or monthly [30]. Furthermore, perceptions of the ability to use the internet and eHealth literacy levels increased significantly after administration of a brief computer and eHealth class (*P*<.05 and *P*<.01, respectively) [27].

### eHealth Literacy and Health Outcomes in People Living With HIV

In this review, 6 of 7 studies examined the associations between eHealth literacy and a variety of health outcomes in PLWH. The 2 studies that measured the relationship between eHealth literacy and HIV-related behavior reported conflicting results. In Blackstock et al [28], higher eHealth literacy was found to be associated with more significant HIV transmission risk behaviors among women living with HIV, including vaginal or anal intercourse without a condom and illicit drug use in the past 30 days, adjusted for income and perceived health status, AOR 3.90 (95% CI 1.05-14.56). The authors suggested the complexities of eHealth literacy across unique social contexts as a possible explanation for the unexpected finding.

In contrast, following an electronically delivered health literacy intervention targeting HIV-related health literacy on medication adherence, participants in Ownby et al [26] self-reported increased knowledge about barriers to adherence and medication misconceptions (*P*=.02) as well as adherence behavioral skills, including using reminders, scheduling medications with other daily activities, and soliciting social support (*P*=.02). Data were collected 3 months apart; however, no control group was included in this study.

Siedner et al [24] examined participant retention in HIV care by measuring attendance at return-to-clinic appointments in accordance with instructions. Following an intervention that involved providing test results through SMS text messages, 60.8% of participants returned to the clinic when provided instructions through SMS text messages [24]. The ability to read a complete sentence on enrollment was independently associated with an accurate identification of the message sent, AOR 4.54 (95% CI 1.42-14.47; *P*=.01), and return to the clinic within 7 days of the first transmitted SMS text message, AOR 3.81 (95% CI 1.61-9.03; *P*=.002) [24]. In addition, the ability to access an SMS text message on enrollment was independently associated with returning to the clinic within 7 days of the SMS text message notification, AOR 4.90 (95% CI 1.06-22.61; *P*=.04) [24].

**Table 3:** Overview of the included studies.

Study	Measurement of eHealth Literacy (Validity or Reliability)	HIV-Related Health Outcome	Main Findings
Blackstock et al, 2016 [28]	eHEALS^a^ Dichotomized at the median (high vs low health literacy; alpha=.88)	HIV transmission risk behaviors, including condomless vaginal or anal intercourse, and any illicit drug use in the previous 30 days	Higher eHealth literacy, AOR^b^ 3.90 (95% CI 1.05-14.56), significantly associated with HIV transmission risk behaviors, adjusted for income and self-perceived health status.
Kim et al, 2015 [29]	Study questions: “Are you able to read?” and “Are you able to write?” (validity or reliability not reported)	Viral suppression (CD4^c^ count), adherence to ART^d^	Literate mobile phone users had lower adherence to ART (84.2% vs 90.6%; *P*=.007) and more favorable perception of utilizing reminders to support the adherence to treatment (57.1% vs 36.7%; *P*<.001) than those who were either illiterate, did not have a mobile phone, or both. There was no difference between literate mobile users and other study participants in the virological suppression.
Krishnan et al, 2015 [30]	Short Test of Functional Health Literacy in Adults (validity or reliability not reported)	ART adherence	No significant differences were found in communication technology use and mHealth acceptance among participants with alcohol use disorders, depression, and suboptimal ART adherence.
Ownby et al, 2012 [26]	TOFHLA^e^<59, inadequate; 60-74, marginal; >75, adequate (validity or reliability not reported)	Medication adherence	Changes in the adherence only approached the statistical significance. Knowledge and behavioral skills increased over the course of the study.
Robinson et al, 2010 [27]	eHEALS (validity or reliability not reported)	HIV-related health outcome not measured	A significant improvement from the baseline to immediately following the intervention was observed in perceived eHealth literacy levels (mean summary score 19 vs 32, *P*<.01) and perceptions of ability to use the internet (*P*<.05).
Siedner et al, 2015 [24]	Participants were asked to read a complete sentence in the local language (validity or reliability not reported)	Retention in care defined as a return to the clinic within 7 days of the first SMS^f^ text message for those with abnormal results or on the date of the scheduled appointment for those with normal results	The ability to read a complete sentence on enrollment was independently associated with accurate identification of the message sent, AOR 4.54 (95% CI 1.42-14.47), and return to the clinic within 7 d of the first transmitted SMS text message, AOR 3.81 (95% CI 1.61-9.03). An ability to access an SMS text message on enrollment was independently associated with returning to the clinic within 7 days of an abnormal SMS text notification, AOR 4.90 (95% CI 1.06-22.61).
Woods et al, 2016 [25]	TOPS^g^; TOHRN^h^; eHEALS; Rapid Estimate of Adult Literacy in Medicine; HIV Knowledge 18; Expanded Numeracy Scale; TOFHLA; Short Assessment of Health Literacy; Newest Vital Sign (validity or reliability not reported)	CD4 count and HIV plasma viral load	Lower TOPS scores were associated with fewer years of education (ρ=.49, *P*=.003), higher HIV viral load (correlation=−.47, *P*=.006), less frequent computer and internet use (*P*<.05) and not owning a smartphone (*P*<.05); lower TOHRN scores were associated with lower education (ρ=.40, *P*=.01), higher HIV viral load (ρ=–.032, *P*=.045), less frequent internet use (*P*<.05), and anxiety related to computer use (*P*<.05).

^a^eHEALS: eHealth Literacy Scale.

^b^AOR: adjusted odds ratio.

^c^CD4: cluster of differentiation 4.

^d^ART: antiretroviral therapy.

^e^TOFHLA: Test of Functional Health Literacy in Adults.

^f^SMS: short message service.

^g^TOPS: Test of Online Pharmacy Skills.

^h^TOHRN: Test of Online Health Records Navigation.

The relationship between eHealth literacy and HIV treatment adherence was mixed. Literacy was inversely associated with ART adherence, which was measured by Kim et al [29] as the self-reported number of missed doses per month (86.4% adherence among literate PLWH with a phone vs 90.6% adherence among not literate PLWH or those with no phone; AOR=1.76; 95% CI 1.12-2.77; *P*=.007). Krishnan et al [30] found no significant differences between patients with optional and suboptimal adherence in their access to communication technology overall; however, a significant difference was observed for mHealth acceptance among participants with and without optimal ART adherence (*P*<.01); for example, participants with poor adherence were less likely to be interested in anonymous internet interaction with a health professional to discuss HIV-related issues compared with participants with optimal adherence (*P*<.001) [30]. Ownby et al [26] attempted to improve the rates of adherence with an electronically delivered health literacy intervention; after this intervention, the adherence increased by 2.3% overall, resulting in the statistical significance among participants who were <95%, <90%, and <85% adherent (*P*=.01,.009,.04, respectively) but not among those in adherence categories of ≤75% [26]. These conflicting results about the relationship between the adherence and eHealth literacy might have been, in part, because of the complexities of measuring the adherence primarily with self-report as well as the nuanced differences between participants exhibiting high- and low-level adherence.

Because only 2 studies assessed participants’ HIV viral load under dissimilar study settings, we were unable to determine the association between eHealth literacy and HIV viral suppression [25,29]. Woods at al [25] reported, among a small sample of 46 HIV-infected participants with and without HIV-associated neurocognitive disorders, poorer performance in Web-based health care navigation tasks was associated with fewer years of education (ρ=.49, *P*=.003), higher plasma HIV viral load (ρ=–.47, *P*=.006), less frequent computer and internet use (*P*<.05), not owning a smartphone (*P*<.05), and higher anxiety related to using a computer (*P*<.05). According to Kim et al [29], in a large-scale study (n=895) with participants having advanced immunosuppression, however, the proportion of participants with an HIV viral load of >1000 copies/mL did not differ between literate phone owners (9%) and phone users who could not read and write (5.7%, *P*=.09).

## Discussion

Although there has been limited reporting on eHealth literacy targeting PLWH, available studies addressing eHealth literacy in PLWH varied in their scope, methodology, and outcomes. The studies included in the systematic review provide some evidence for the role of eHealth literacy in relation to diverse HIV-related health outcomes, including HIV transmission risk, retention in care, treatment adherence, and virological suppression. Even though eHealth literacy was generally high and majority of those individuals included in the samples were receptive to the use of SMS text messaging communication [29], findings were mixed with instances of eHealth literacy both promoting as well as hindering health outcomes.

In descriptive studies, eHealth literacy was either inversely associated with HIV transmission prevention behaviors, ART adherence, or viral load [25,28,29] or unrelated to the adherence [30]. In contrast, eHealth literacy showed promise in promoting increased HIV knowledge and HIV-related behavioral skills, return visits when linked to care, and in bolstering the adherence in studies using quasi-experimental or experimental designs [24,26]. Each of these factors is critical in maintaining positive outcomes related to knowledge and behaviors [26].

Negative outcomes in retention in care and treatment adherence may be attributed to general literacy challenges and access to phones, laptops, and desktop devices [24,30]. In addition, the findings may be attributable to methodological biases associated with the studies included in the review. Specifically, although 1 RCT [24] and 1 of 3 quasi-experimental studies [25] had strengthened the validity of causal inferences by comparing control and intervention groups, the baseline differences between participants’ characteristics in both groups were unclearly reported. In addition, 2 quasi-experimental studies [26,27] lacked a comparison group to determine pre-post intervention effects. Thus, the relationships among eHealth literacy and linkage to care [24], Web-based health care navigation tasks [25], medication adherence [26], and internet health literacy and confidence [27] could not be attributed to the potential causal effect. Moreover, 2 of 7 studies did not use a validated standard measure of eHealth literacy but collected participants’ basic literacy skills [24,29]. Self-reported literacy may result in not only the limited accuracy of data collected but also social desirability bias [38].

This review has revealed several gaps in the existing evidence base; gaps that collectively point to what we argue should be key parts of the eHealth literacy research agenda going forward. The most important gap and a critical focus of future research is the use of validated instruments to measure eHealth literacy, which do not appear in these studies. Much of the research we reviewed used some form of eHealth literacy assessment but with no evidence of validity and reliability or proxy measures for eHealth literacy. Future eHealth literacy research should adopt more rigorous instrumental approaches to addressing eHealth literacy as a new way of promoting and facilitating self-management in PLWH. In addition, there exists a limited explanation of definitions of eHealth literacy used in the literature. Hence, the selection of study instruments was minimally justified within the reviewed studies, highlighting the need for adopting a validated eHealth literacy framework to better understand and promote healthy behaviors and outcomes of PLWH. Finally, this review highlighted a critical methodological gap and area for future improvement—the need for ensuring a rigorous study design with adequate sample size, use of validated eHealth literacy measures and theoretical framework, and the use of diverse study samples of PLWH; for example, because >90% of adolescents and young adults use the internet daily [39], youth needs to receive more attention in eHealth literacy research as they may have a different level of eHealth literacy than older adults. Finally, because qualitative studies or mixed-methods studies provide diversified, in-depth perspectives, the combination of quantitative and qualitative data would contribute to the development of a complete understanding of the eHealth literacy among PLHW.

Although the strengths of this review’s design included its inclusive search strategy that ensured extensive coverage, standardized data extraction, and iterative analysis, there are several limitations. First, despite our expanded search criteria, only a small number of studies met the inclusion criteria because of a lack of published studies. Second, the heterogeneity in the quality and quantity of data reported in the studies included in the review. Finally, we were unable to include studies in languages other than English, thereby limiting the generalizability of our findings.

In conclusion, the importance of eHealth literacy among PLWH has only recently begun to be addressed. In the areas of HIV transmission risk, retention in care, treatment adherence, and virological suppression, the role of eHealth literacy remains partially understood. Understanding the role of eHealth literacy among PLWH is an essential next step in self-management of HIV and AIDS. Avenues to pursue in the role of eHealth literacy and PLWH should include the development and use of standardized eHealth literacy measures. Additionally, examining the role of eHealth literacy longitudinally from prevention to viral suppression could yield knowledge regarding at what point, from diagnosis through management, are the best points to intervene with eHealth literacy strategies. Finally, elucidating the other factors that potentially contribute to eHealth literacy, such as access and general literacy, could yield valuable findings going forward.

## Appendix

Multimedia Appendix 1 Search strategy.

## Abbreviations

AOR: adjusted odds ratio
ART: antiretroviral therapy
CD4: cluster of differentiation 4
eHEALS: eHealth Literacy Scale
IQR: interquartile range
PLWH: people living with HIV
RCT: randomized controlled trials
SMS: short message service
TOFHLA: Test of Functional Health Literacy in Adults
TOHRN: Test of Online Health Records Navigation
TOPS: Test of Online Pharmacy Skills
