# Supplementary material for: eHealth Literacy in People Living with HIV: Systematic Review
Source: JMIR Public Health Surveill. 2018 Sep 10;4(3):e64. doi: 10.2196/publichealth.9687 (PMC6231824; doi:10.2196/publichealth.9687)
Supplement: Multimedia Appendix 1 [file publichealth_v4i3e64_app1.pdf]

**Appendix 1. Search Strategy**			
**Database**	Search Terms	Search Date	Number of Citations Retrieved
**PubMed**	(((((hiv infections [mh] OR hiv [mh] OR hiv [tw] OR hiv-1[tw] OR hiv-2[tw] OR hiv1[tw] OR hiv2[tw] OR hiv infect*[tw] OR "human immunodeficiency virus" [tw] OR "human immunedeficiency virus" [tw] OR "human immuno-deficiency virus" [tw] OR "human immune-deficiency virus" [tw] OR ((human immun*) AND (deficiency virus[tw])) OR "acquired immunodeficiency syndrome" [tw] OR "acquired immunedeficiency syndrome" [tw] OR "acquired immuno-deficiency syndrome" [tw] OR "acquired immune-deficiency syndrome" [tw] OR ((acquired immun*) AND (deficiency syndrome[tw])))))))<br>AND<br>("Telemedicine"[Mesh] OR "mobile health" OR "ehealth" OR "mhealth" OR "telehealth")<br>AND<br>("Health Literacy"[Mesh] OR "Consumer Health Information"[Mesh] OR "health literacy" OR "health literate" OR literacy [tiab] OR literate [tiab] OR illiterate [tiab])	April 27, 2017	19
**EMBASE**	'human immunodeficiency virus infection'/exp OR 'human immunodeficiency virus infection' OR 'human immunodeficiency virus'/exp OR 'human immunodeficiency virus' OR 'acquired immune deficiency syndrome'/exp OR 'acquired immune deficiency syndrome' OR 'hiv':ab,ti OR 'hiv-1':ab,ti OR 'hiv-2':ab,ti OR 'hiv1':ab,ti OR 'hiv2':ab,ti OR 'human immunodeficiency virus':ab,ti OR 'human immunedeficiency virus':ab,ti OR 'human immuno-deficiency virus':ab,ti OR 'human immune-deficiency virus':ab,ti OR (human NEAR/4 immune* AND deficiency NEAR/4 virus) OR 'acquired immunodeficiency syndrome':ab,ti OR 'acquired immunedeficiency syndrome':ab,ti OR 'acquired immuno-deficiency syndrome':ab,ti OR 'acquired immune-deficiency syndrome'/exp OR 'acquired immune-deficiency syndrome' OR (acquired NEAR/4 immun* AND deficiency NEAR/4 syndrome)<br>AND<br>'health literacy'/exp OR (health NEAR/3 (literacy OR literate OR illiterate)):ti,ab OR literate:ti,ab OR	April 27, 2017	17

	literacy:ti,ab OR illiterate:ti,ab<br>AND<br>'telehealth'/exp OR 'mobile health':ti,ab OR 'ehealth':ti,ab OR 'mhealth':ti,ab OR 'telehealth':ti,ab OR 'telemedicine':ti,ab		
**CINAHL**	((MH "HIV Infections+") OR "hiv" OR (MH "Human Immunodeficiency Virus+") OR hiv*) OR (human immunodeficiency virus OR humanimmunedeficiency virus oR human immuno-deficiency virus OR human immune-deficiency virus OR (human immun*) N3 (deficiency virus)) OR (acquired immunodeficiency syndrome OR acquired immune-deficiency syndrome OR acquired immunedeficiency syndrome OR acquired immuno-deficiency syndrome OR (acquired immune) N3 (deficiency syndrome))<br>AND<br>((MH "Health Literacy") OR (MH "Consumer Health Information")) OR (health N3 (literate OR literacy OR illiterate)) OR (literacy OR literate OR illiterate)<br>AND<br>(MH "Telehealth+") OR ("mobile health" OR "ehealth" OR "mhealth" OR "telehealth" OR "telemedicine")	April 27, 2017	5
**Scopus**	(((TITLE-ABS-KEY (hiv* OR "hiv infection" OR "hiv infections" OR (human W/3 immune* AND deficiency W/3 virus) OR "acquired immunodeficiency syndrome" OR "acquired immunedeficiency syndrome" OR "acquired immuno-deficiency syndrome" OR "acquired immune-deficiency syndrome" OR (acquired W/3 immun* AND deficiency AND near/3 AND syndrome)) OR TITLE-ABS-KEY (acquired AND immun* W/3 (virus OR syndrome)))))<br>AND<br>((TITLE-ABS-KEY (health W/3 literacy) OR TITLE-ABS-KEY (health W/3 literate) OR TITLE-ABS-KEY (health W/3 illiterate) OR TITLE-ABS-KEY (literacy OR literate OR illiterate)))<br>AND<br>("ehealth" OR "mhealth" OR telemedicine OR "telehealth" OR "tele-health")	April 27, 2017	40
**Web of Science**	**TOPIC:** (((hiv* OR "hiv infection" OR "hiv infections" OR (human NEAR/3 immune* AND deficiency NEAR/3 virus) OR "acquired immunodeficiency syndrome" OR "acquired immunedeficiency syndrome" OR "acquired immuno-deficiency syndrome" OR "acquired immune-deficiency syndrome" OR (acquired NEAR/3 immun*	April 27, 2017	15

	AND deficiency NEAR/4 syndrome))))<br>*AND*<br>**TOPIC:** ((health NEAR/3 (literacy OR literate OR illiterate) OR literate OR literacy OR illiterate))<br>*AND*<br>**TOPIC:** (("ehealth" OR "mhealth" OR telemedicine OR "telehealth" OR "tele-health"))<br>**Timespan:** All years. **Indexes:** SCI-EXPANDED, SSCI, A&HCI, CPCI-S, CPCI-SSH, BKCI-S, BKCI-SSH, ESCI, CCR-EXPANDED, IC.		
**Cochrane**	#1 MeSH descriptor: [HIV Infections] explode all trees<br>#2 MeSH descriptor: [HIV] explode all trees<br>#3 hiv* or hiv near/3 infect* or ((human or acquir*) near/3 immune* near/3 (virus or syndrome))<br>#4 #1 or #2 or #3<br>#5 MeSH descriptor: [Consumer Health Information] explode all trees<br>#6 MeSH descriptor: [Health Literacy] explode all trees<br>#7 health near/3 (literate or literacy or illiterate) or literacy or literate or illiterate<br>#8 #5 or #6 or #7<br>#9 #4 and #8<br>#10 MeSH descriptor: [Telemedicine] explode all trees<br>#11 ("mobile health" or "ehealth" or "mhealth" or "telehealth" or "telemedicine")<br>#12 #10 or #11<br>#13 #9 and #12	April 27, 2017	2
